# Metabolic programming of adipose tissue structure and function in male rat offspring by prenatal undernutrition

**DOI:** 10.1186/1743-7075-11-50

**Published:** 2014-10-18

**Authors:** Nichola Thompson, Korinna Huber, Mirijam Bedürftig, Kathrin Hansen, Jennifer Miles-Chan, Bernhard H Breier

**Affiliations:** Discipline of Physiology, School of Medical Sciences, Faculty of Health Sciences, The University of Adelaide, Adelaide, SA 5005 Australia; Department of Physiology, University of Veterinary Medicine, 30173 Hannover, Germany; Institute of Physiology, Department of Medicine, University of Fribourg, 1700 Fribourg, Switzerland; Institute of Food, Nutrition and Human Health, College of Health, Massey University, Albany Campus, Auckland, 1142 New Zealand

**Keywords:** Subcutaneous adipose tissue, Retroperitoneal adipose tissue, Insulin response, Beta-adrenergic response, Prenatal undernutrition

## Abstract

**Background:**

A number of different pathways to obesity with different metabolic outcomes are recognised. Prenatal undernutrition in rats leads to increased fat deposition in adulthood. However, the form of obesity is metabolically distinct from obesity induced through other pathways (e.g. diet-induced obesity). Previous rat studies have shown that maternal undernutrition during pregnancy led to insulin hyper-secretion and obesity in offspring, but not to systemic insulin resistance. Increased muscle and liver glycogen stores indicated that glucose is taken up efficiently, reflecting an active physiological function of these energy storage tissues. It is increasingly recognised that adipose tissue plays a central role in the regulation of metabolism and pathophysiology of obesity development. The present study investigated the cell size and endocrine responsiveness of subcutaneous and visceral adipose tissue from prenatally undernourished rats. We aimed to identify whether these adipose tissue depots contribute to the altered energy metabolism observed in these offspring.

**Methods:**

Adipocyte size was measured in both subcutaneous (ScAT) and retroperitoneal adipose tissue (RpAT) in male prenatally *ad libitum* fed (AD) or prenatally undernourished (UN) rat offspring. Metabolic responses were investigated in adipose tissue explants stimulated by insulin and beta_3_ receptor agonists *ex vivo.* Expression of markers of insulin signalling was determined by Western blot analyses. Data were analysed by unpaired t-test or Two Way ANOVA followed by Fisher’s PLSD post-hoc test, where appropriate.

**Results:**

Adipocytes in offspring of undernourished mothers were larger, even at a lower body weight, in both RpAT and ScAT. The insulin response of adipose tissue was reduced in ScAT, and statistically absent in RpAT of UN rats compared with control. This lack of RpAT insulin response was associated with reduced expression of insulin signalling pathway proteins. Adrenergic receptor-driven lipolysis was observed in both adipose depots; however insulin failed to express its anti-lipolytic effect in RpAT in both, AD and UN offspring.

**Conclusions:**

Metabolic dysregulation in offspring of undernourished mothers is mediated by increased adipocyte size and reduced insulin responsiveness in both ScAT and especially in RpAT. These functional and morphological changes in adipocytes were accompanied by impaired activity of the insulin signalling cascade highlighting the important role of different adipose tissue depots in the pathogenesis of metabolic disorders.

## Background

The global escalation of obesity is currently one of the world’s largest health concerns. Obesity contributes to a chronic pro-inflammatory state and to deterioration of glucose and lipid metabolism. Metabolic dysregulation increases an individual’s risk of developing a range of non-communicable diseases including type 2 diabetes and cardiovascular disease. Known contributing factors for the development of obesity include imbalances in pathways of glucose and lipid metabolism as a consequence of extrinsic and intrinsic factors - such as variations in quantity and quality of nutrition, sedentary lifestyle and genetic predisposition [1–3].

Nutritional perturbations during early life increase the long-term susceptibility to obesogenic environmental factors, including hypercaloric diets and sedentary behaviour [4]. In previous studies we developed a rat model of prenatally-induced obesity by restricting maternal food intake during pregnancy to 30% of the amount of food eaten by the *ad libitum* fed pregnant dams. The prenatally undernourished (UN) offspring are shorter and lighter at birth and remain shorter throughout life compared with prenatally adequately (AD) nourished offspring. However, UN offspring show catch-up growth in terms of body weight increases after weaning and develop metabolic abnormalities in adult life that include obesity, hyperinsulinemia, hyperleptinemia, and hypertension [5, 6]. Interestingly, these rodents develop a distinct metabolic phenotype in adulthood with increased adipose tissue fat accretion but maintained whole body insulin sensitivity measured by hyperinsulinemic-euglycemic clamp [6].

The maintained insulin sensitivity in UN offspring is further supported by the presence of normal levels of liver and muscle fat content [5, 6]. While the plasma glucose, triglyceride and insulin levels were enhanced in our previous studies; intriguingly, glycogen stores in liver and muscle were significantly increased. The increase of glycogen stores in UN offspring was not based on changes in hepatic gluconeogenesis, as indicated by maintained gene expression of pyruvate carboxylase and phosphoenolpyruvate carboxykinase in UN offspring [6, 7]. In contrast, the opposite metabolic state is observed in rodents, when obesity is induced by feeding a diet high in fat and carbohydrate [6]. It is well established that white adipose tissue depots actively contribute to energy storage and release, and there is growing evidence of major ontogenetic differences between visceral and subcutaneous adipose tissue [8]. We therefore hypothesise that a prenatal pathway to obesity, as observed in UN offspring, may have set in train distinct metabolic and energy storage characteristics in subcutaneous and retroperitoneal adipose tissues [6].

Adipose tissues have a strong influence on glucose and lipid metabolism and systemic insulin sensitivity through the storage of energy as triglycerides and by secreting a variety of adipokines [9]. Moreover, different adipose tissue depots have distinct physiological and metabolic properties. For example, central fat within the abdominal cavity, visceral adipose tissue, is increasingly considered to play a stronger role in driving metabolic dysregulation and inflammation than subcutaneous adipose tissue located underneath the skin [10]. We hypothesise that specific adipose tissue depots may contribute to the previously observed shift in glucose flux towards liver and muscle in UN offspring through reduced glucose uptake. In the present report, we test this hypothesis by conducting *ex vivo* studies in both subcutaneous and visceral retroperitoneal adipose tissue depots from male UN offspring employing a well characterised model of metabolic programming [5–7]. We examined cell sizes, metabolic responses to insulin, and catecholamine stimulation *ex vivo* and the expression of markers of insulin signalling and lipolytic pathways in both subcutaneous (ScAT) and retroperitoneal adipose tissue (RpAT).

## Methods

### Experimental design

All animal experiments were conducted under the control and authorisation of the Lower Saxony State Office for Consumer Protection and Food Safety, according to the German Animal Welfare Law (reference no. 33.9-42502-04-09/1731). 25 male and 50 virgin female outbred Wistar rats (~270 g body weight (bwt)) were mated. Successful mating was confirmed by sperm detection in the vaginal smear, and pregnant dams were individually housed with *ad libitum* access to water. Pregnant dams were randomly subdivided into two experimental groups. Half were given *ad libitum* access to chow (standard chow, Altromin, Lage, Germany). The other half received 30% of the food intake of their *ad libitum*-fed counterparts, calculated daily throughout pregnancy. Body weights of the pregnant rats were recorded daily until parturition. Approximately 12 hours following birth all offspring were weighed and measured, and cross-fostered to *ad libitum* nourished mothers. Litter size was reduced to 5 rats per mother to ensure *ad libitum* milk intake for all pups. After weaning, male prenatally *ad libitum*-fed (AD) and undernourished (UN) offspring (32 rats/group) were housed in pairs with free access to standard chow and water. Food intake was monitored weekly for each cage and individual food intake calculated. Throughout the experiment, all rats were housed at 23 ± 1°C and 60% humidity with a day-night-cycle of 12 h and wood shavings as bedding material.

### Sample collection

At 9 months of age, following an overnight fast, male AD and UN offspring were anaesthetised with CO_2_, body weight and length were measured, and then rats were killed by decapitation. Trunk blood was collected into heparinised and serum tubes, centrifuged at 2,000 × g for 10 min; and plasma and serum aliquots were removed and stored at -80°C until analysis. Subcutaneous adipose tissue (ScAT) samples were collected from the inguinal region and retroperitoneal adipose tissue (RpAT) was obtained from the abdominal cavity, avoiding contamination with the perirenal fat. Connective tissue and blood vessels were removed, then samples were weighed and washed in ice-cold saline. Fresh adipose tissue samples were collected for the *ex vivo* incubation studies, and additional tissue aliquots were snap-frozen in liquid nitrogen and stored at -80°C.

### Chemicals and antibodies

Analytical grade biochemicals, glycerol assay kits and hormones/agonists were obtained from Sigma-Aldrich Inc. (St. Louis, MO) and medium from Invitrogen (Carlsbad, CA) unless otherwise specified. Reagents and apparatus for SDS-PAGE and immunoblotting were from BioRad (Hercules, CA). Primary antibodies for Western blotting were obtained from Cell Signaling Technology (Danvers, MA; mammalian target of rapamycin (mTOR), hormone-sensitive lipase (HSL)), Santa Cruz Biotechnology (Santa Cruz, CA; insulin receptor beta subunit (InsRβ), phosphatidylinositol-3-kinase (PI3K) and protein kinase C zeta (PKCζ)) and Chemicon International Inc. (Temecula, CA; perilipin).

### Ex vivo incubation studies

#### General method

Adipose tissue pieces (20-25 mg) were incubated in 0.5 ml Dulbecco´s modified Eagle medium (DMEM) at 37°C and agitated at 400 rpm (3 mm amplitude; Eppendorf Thermomixer). Adipose tissue incubations were performed in triplicate for each tissue. Following the incubation period, the medium was removed and used for spectrophotometric measurement of glucose (hexokinase/glucose-6-phosphate dehydrogenase assay; D-glucose assay kit; r-biopharm, Boehringer, Mannheim, Germany; sensitivity 0.4 mg/l) and glycerol (glycerol-3-phosphate oxidase (GPO) method; Free glycerol assay kit; sensitivity 0.03 mmol/l) concentrations in triplicate.

### Insulin-stimulated glucose uptake assay

Tissue samples were incubated for 2.5 h in DMEM supplemented with 1% BSA and glucose to a concentration of 0.225 g/l with or without insulin (10 mU/ml; recombinant human insulin, Sigma). Insulin concentrations and incubation times were determined in preliminary experiments. Post-incubation medium was collected and glucose concentration measured. The decrease in medium glucose concentration in control and insulin-supplemented incubations was considered equal to the glucose uptake by the adipose tissue (0.225 g/l before incubation – × g/l after incubation = glucose uptake). Glucose uptake was then corrected for adipose tissue weight (final glucose uptake rate in μg/mg/2.5h).

### Adrenergic receptor-induced glycerol release

Tissue samples were incubated for 1 h in DMEM containing 0.5% fatty acid-free BSA (control media). To assess the response of tissue explants to adrenergic receptor stimulation the control media was supplemented with BRL, a beta_3_ receptor agonist (BRL 37344; 50 ng/ml, Sigma) or BRL plus insulin (2.5 mU/ml recombinant human insulin, Sigma). Post incubation medium was collected and glycerol concentration measured. The glycerol concentration of the media was corrected for adipose tissue weight.

### Blood sample analyses

Plasma glucose concentration was measured by the spectrometric glucose oxidase/peroxidase-anti-peroxidase method (mtidiagnostics, Idstein, Germany; sensitivity 0.11 mmol/l)), and plasma triglyceride concentration by the spectrometric GPO method (WAK-Chemie, Steinbach, Germany, sensitivity 0.05 mmol/l).

### Determination of adipocyte sizes

Snap frozen RpAT and ScAT were sectioned (10 μm) on a cryostat at -40°C (Cryostat Jung CM3, Leica, Switzerland). 6-10 random sections of each tissue sample were mounted on Superfrost slides and stained with hematoxylin and eosin. Cells were visualised using a light microscope fitted with a digital camera (Leica, Switzerland) at 10x magnification. 5 areas per section were digitally photographed. The individual areas (μm^2^) of 400 adipose cells in total were measured per adipose tissue depot using Leica Application Suite software for histometry (Leica, Switzerland).

### Western Blot determination of marker proteins of insulin signalling and lipolysis

30- 50 mg of frozen adipose tissue was homogenised in 1 ml of prechilled homogenisation buffer (50 mmol/l HEPES, pH 7.4, 0.1% Triton X-100, 4 mmol/l EGTA, 10 mmol/l EDTA, 100 mmol/l β-glycerophosphate, 15 mmol/l tetrasodium pyrophosphate, 5 mmol/l sodium orthovanadate, 25 mmol/l sodium fluoride, and protease inhibitors (Roche, Mannheim, Germany); Homogenates were shaken for 2 h at 4°C and subsequently passed 20x through a syringe with a 26 gauge needle followed by 20x with a 22 gauge needle. Homogenates were centrifuged at 90 g for 10 min at 4°C to remove the fat. Fat-free homogenates were obtained and aliquots were stored at -20°C until further analysis. Protein concentrations of the homogenates were measured according to Bradford (SERVA protein quantification kit). Samples in loading buffer (50 mmol/l TrisHCL, pH 6.8, 10% glycerol, 2% sodiumdodecylsulfate, 0.1% bromphenol blue, 4% mercaptoethanol) were heat-denatured prior to loading 10 μg per lane on a 5% stacking/8.1% separation gel. Electrophoresis was performed according to Laemmli [11]; separated proteins were transferred onto nitrocellulose membranes by tank blotting. Detection of specific proteins was performed after blocking the membranes for 2 h in 10% fat free milk/PBST at RT. Membranes were incubated overnight at 4°C with rat-specific primary antibodies at the given concentrations: InsRβ, 1:50; PI3K, 1:200; PKCζ 1:200; mTOR 1:200; HSL 1:1000; perilipin 1:10000. Detection of the primary antibodies was performed using secondary anti-rabbit-HRP antibodies: for either 1 h (PI3K 1:2000, PKCζ 1:2000 HSL 1:2000, perilipin 1:2000), 1.5 h (InsRβ 1:25000), or 2 h (mTOR 1:25000) at RT. Following washing, membranes were incubated with Pierce (Thermo Scientific, Rockford, IL; InsR, mTOR) and Lumiglo (KPL, Gaithersburg, ML; PI3K, PKCζ substrate. Equal loading of lanes was checked by staining the membranes with Indian ink. After band detection, extent of expression was quantified by densitometry with Quantity One software (BioRad).

### Statistics

Data are presented as mean ± SEM; Data were statistically analysed by unpaired t-test or a repeated measures Two Way ANOVA followed by Fisher´s PLSD post-hoc test, where appropriate (Statview software, SAS Institute Inc., Cary, NC, USA). Linear relationships between body weight and variables of glucose metabolism were detected by linear regression analysis. Details of the specific sample subset for each experiment and the statistical evaluation of each data set are given in the text, tables, and in the figure legends. A p-value ≤0.05 was set to be significant.

## Results

### Metabolic status of the AD and UN offspring

**Figure 1:**
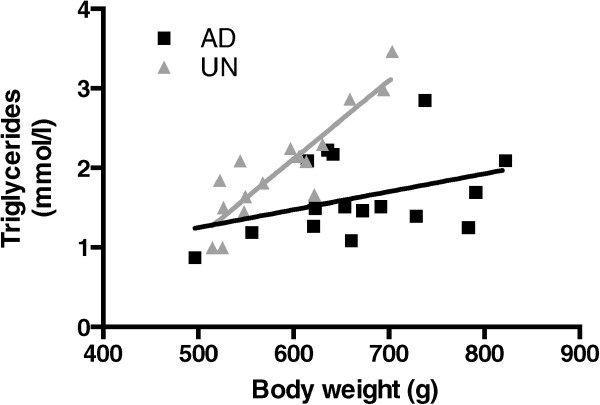
**Plasma triglyceride concentrations related to body weight in prenatally adequately nourished AD (black boxes, n = 16) and prenatally undernourished UN (grey triangles, n = 16) offspring.** Slope of linear regression line of UN data analysis is significantly different from zero in UN (grey triangle) with p < 0.0001 and the goodness of fit is demonstrated by a coefficient of determination r^2^ = 0.79.

As observed in our previous studies [5–7, 12], maternal undernutrition during pregnancy has a profound influence on metabolic regulation in offspring, throughout their postnatal development. The UN offspring were lighter and shorter than their AD counterparts at the end of the experiment. Litter size was similar at birth in both *ad libitum* and food restricted groups with 15 ± 3 and 13 ± 4 pups per litter, respectively. Animal data are presented in Table 1. Daily food intake per gram of body weight was similar in both AD and UN offspring. Plasma glucose concentrations did not differ between the two groups. However, circulating triglyceride levels were increased in UN offspring compared with AD offspring. Furthermore, plasma triglyceride concentrations showed a strong positive relationship with body weight in UN offspring only, with much higher triglyceride concentrations (p < 0.01) at higher body weights (Figure 1).

**Table 1:** **Animal data and blood variables of offspring of** ***ad libitum*** **-fed (AD) and undernourished (UN) mothers**

Variable	Unit	AD	UN	P value ^3^	n/group
Body weight^1^	g	647 ± 14	590 ± 13	<0.01	32
Body length^1^	cm	28.5 ± 0.2	27.4 ± 0.1	<0.001	32
Food intake^2^	g/d/rat	29.9 ± 0.6	28.3 ± 0.5	<0.05	32
Food intake/g bwt	g/g	0.047 ± 0.001	0.049 ± 0.001	n.s.	32
Glucose	mmol/l	8.71 ± 0.46	8.92 ± 0.42	n.s.	32
Triglycerides	mmol/l	1.66 ± 0.11	2.04 ± 0.12	<0.05	32
RpAT	g	5.69 ± 0.37	5.65 ± 0.42	n.s	32
RpAT % bwt	%	0.87 ± 0.04	0.93 ± 0.05	n.s	32

Values are given as mean ± SEM.

^1^Body weight (bwt) and length measured at the end of the experiment (9 months of age).

^2^Mean food intake per rat over the whole experimental period was calculated from the measured food intake per cage (2 rats/cage).

^3^Data were statistically analysed by unpaired student’s t-test.

### Adipose tissue cell size

**Figure 2:**
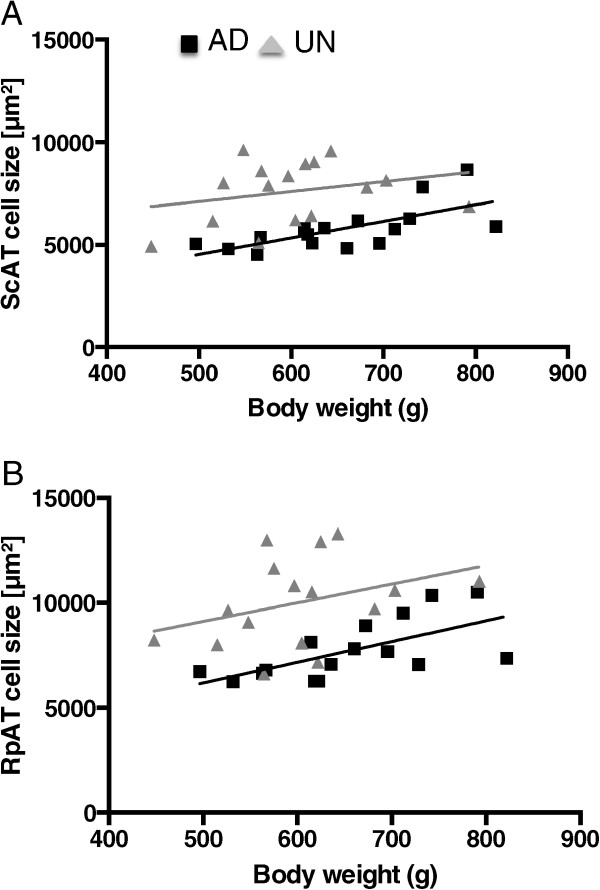
**Cell sizes of subcutaneous (A; ScAT) and retroperitoneal (B; RpAT) adipose tissues related to body weight in prenatally adequately nourished AD (black boxes, n = 16) and prenatally undernourished UN (grey triangles, n = 16) offspring.** Slopes of linear regression lines of AD data analyses are significantly different from zero with p < 0.01 (in ScAT and RpAT) and the goodness of fit is demonstrated by a coefficient of determination r^2^ = 0.46 in ScAT and r^2^ = 0.401 in RpAT. Analyses of UN data in ScAT and RpAT revealed no significant relationship between cell size and body weight.

Adipocyte mean, minimum and maximum cell sizes were significantly higher in UN offspring in both the ScAT and the RpAT depot (Table 2). In general, RpAT had the largest mean, minimum and maximum cell sizes in both AD and UN offspring with largest cell areas exceeding 30,000 μm^2^ in UN offspring. While adipocyte sizes in both RpAT and ScAT of AD rats showed a significant positive correlation with body weight this positive correlation was not observed in UN rats (Figure 2). Thus, this study identifies larger adipocytes at a lower body weight in UN offspring, which is a marker of early adipocyte hypertrophy. There were no differences in the weight of retroperitoneal adipose tissue depots between the two experimental groups either as tissue weight or when calculated as a percentage of body weight (Table 1).

**Table 2:** **Adipose tissue cell sizes of rat offspring of** ***ad libitum*** **-fed (AD) and undernourished (UN) mothers**

	AD		UN		Two Way ANOVA ^1^		
(μm ^2^)	ScAT	RpAT	ScAT	RpAT	PN	AT	I
Mean	5779^A^	7709^B^	7606^B^	10021^C^			
cell size	±274	±353	±373	±519	P < 0.001	P < 0.0001	n.s.
Minimum	1832^A^	2352^B^	2743^B^	3349^C^			
cell size^2^	±132	±189	±199	±282	P < 0.001	P < 0.01	n.s.
Maximum	15525^A^	23462^B^	19053^B^	34269^C^			
cell size^2^	±1344	±1961	±932	±1848	P < 0.001	P < 0.001	P < 0.01

Values are given as mean ± SEM; n = 16/group; ScAT = subcutaneous adipose tissue, RpAT = retroperitoneal adipose tissue.

^1^Factor PN = prenatal nutrition; factor AT = adipose tissue; I = interaction; Means marked with different upper case letters (A, B and C) are significantly different with at least p<0.05; Fisher´s PLSD posthoc test).

^2^Smallest and largest cells of each animal were analysed.

### Glucose disposal in different adipose tissue depots in AD and UN offspring

**Figure 3:**
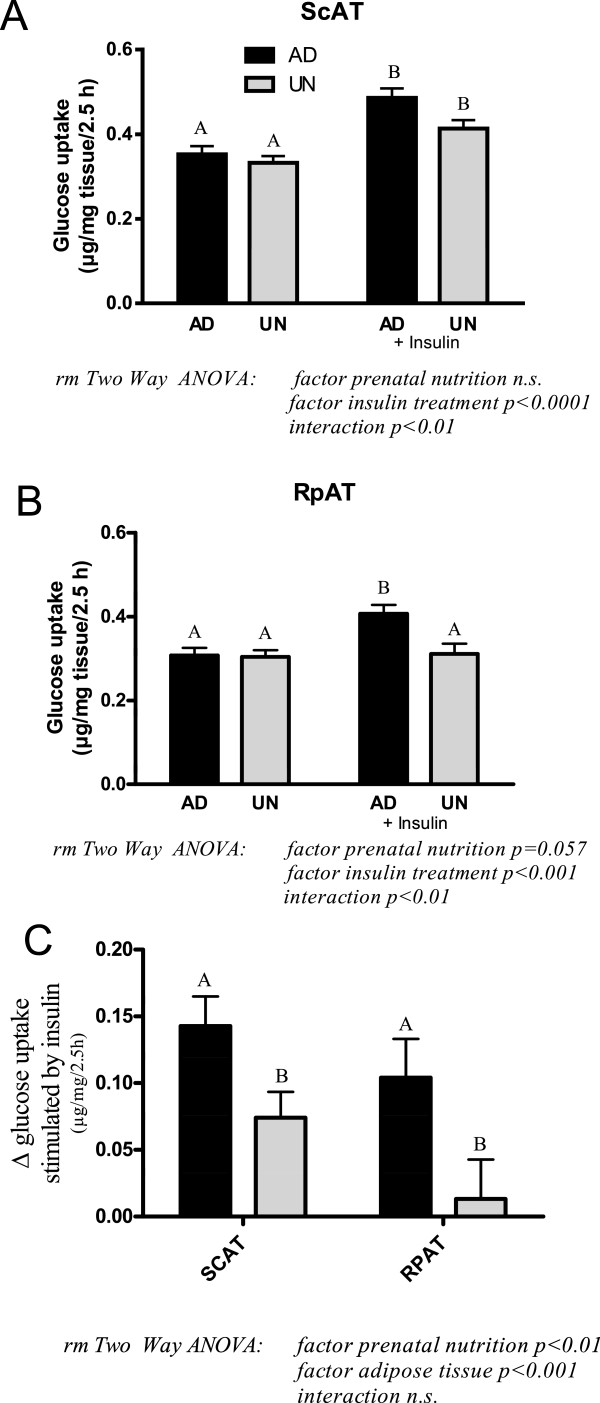
**Insulin-stimulated glucose uptake in subcutaneous (ScAT; A) and retroperitoneal (RpAT; B)** ***ex vivo*** **adipose tissue explants. C** shows the insulin-dependent increase in glucose uptake in adipose tissue explants. Data of **A, B** and **C** are expressed as means ± SEM, n = 16 rats per group. Bars with different upper case letters are significantly different with p < 0.0001 **(A)**, p < 0.001 **(B)** and p < 0.01 **(C)**.

Results of the glucose uptake in *ex vivo* adipose tissue explants are shown in Figure 3A-C. In ScAT, insulin increased glucose uptake in both AD and UN offspring but the magnitude of insulin-stimulated glucose uptake was lower in UN offspring as indicated by a statistically significant interaction. Insulin stimulated glucose uptake in RpAT from AD offspring, in contrast insulin insensitivity was observed in UN offspring RpAT. The insulin-dependent increase in glucose uptake in adipose tissue explants was lower in RpAT compared with ScAT, with the UN group RpAT showing clearly and significantly reduced response (Figure 3C).

**Figure 4:**
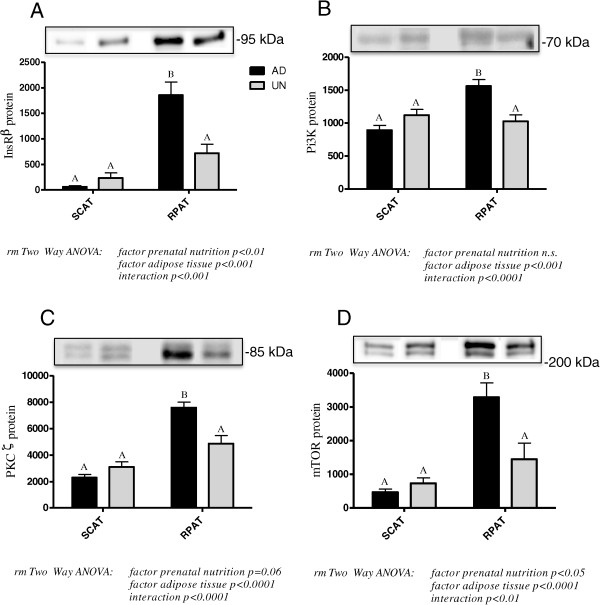
**Protein expression of key components of insulin signalling pathways in subcutaneous (ScAT) and retroperitoneal (RpAT) adipose tissues of prenatally adequately nourished AD and prenatally undernourished UN rat offspring.** Data of **A-D** are expressed as means ± SEM arbitrary units, ScAT n = 16/group, RpAT n = 14/group (due to technical reasons). Bars with different upper case letters are significantly different with p < 0.001 **(A-C)** and p < 0.01 **(D)**. For each protein, representative Western Blots are shown.

Protein expression of markers of insulin signalling, InsRβ, PI3K, PKCζ and mTOR, are presented in Figure 4A-D. In ScAT, comparable levels of these marker proteins were expressed in both AD and UN offspring. In contrast, in RpAT the expression of all insulin signalling marker proteins was significantly lower in UN offspring compared to the AD control group, identifying a down regulation of insulin signalling pathways with prenatal undernutrition. These data are in clear agreement with the lack of insulin-stimulated glucose uptake observed in RpAT (Figure 3C) from UN offspring in the *ex vivo* studies discussed above.

### Lipolytic capacity of different adipose tissue depots in AD and UN offspring

**Figure 5:**
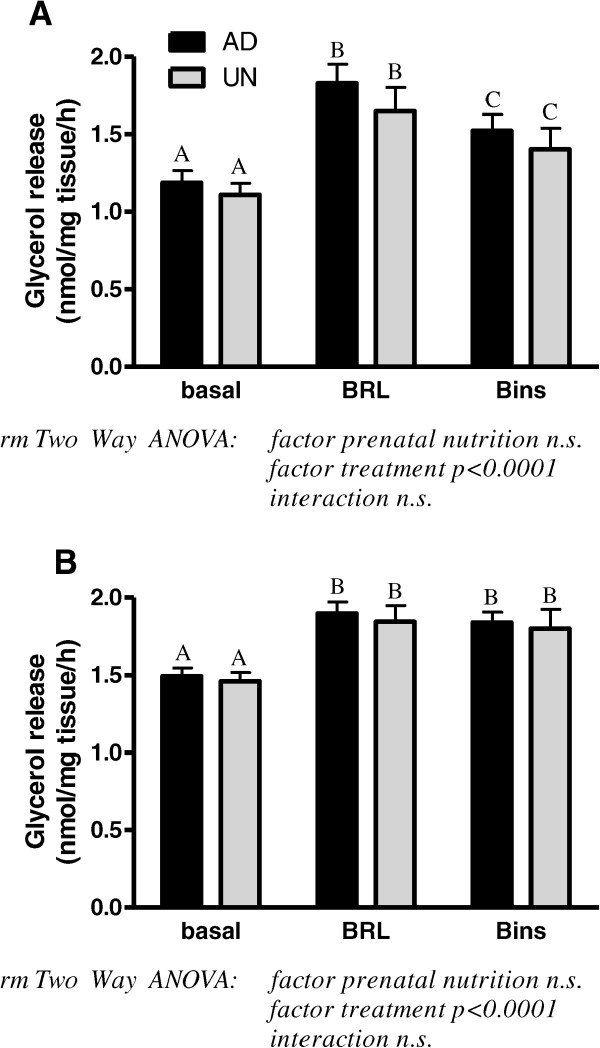
**Lipolytic response as assess by glycerol release to beta**_**3**_ **-adrenergic stimulation in subcutaneous (ScAT, A) and retroperitoneal (RpAT, B) adipose tissue explants** ***ex vivo*** **of prenatally adequately nourished AD and prenatally undernourished UN rat offspring.** Basal = glycerol release following incubation in media, BRL = glycerol release following incubation in media supplemented with beta_3_-adrenergic receptor ligand, Bins = glycerol release following incubation in media supplemented with beta_3_-adrenergic receptor ligand and insulin. Data of **A** and **B** are expressed as means ± SEM, n = 16/group. Bars with different upper case letters are significantly different with p < 0.0001.

Basal glycerol release in adipose tissues was similar in AD and UN offspring but the RpAT had consistently significantly higher values in comparison with ScAT (p < 0.0001, paired t-test). Concomitantly, levels of HSL and perilipin protein, both key factors involved in lipolysis, were significantly higher in RpAT of both, AD and UN offspring (HSL - ScAT: AD 482.3 ± 51, UN 566.3 ± 36; RpAT: AD 700.6 ± 29, UN 691.3 ± 32 (p < 0.0001); perilipin - ScAT: AD 113.6 ± 12, UN 129.8 ± 70; RpAT: AD 174.1 ± 83, UN 169.6 ± 11 (p < 0.0001)). However, prenatal undernutrition did not influence the HSL and perilipin expression in adult life. Beta_3_-adrenergic stimulation of lipolysis in adipose tissues was observed in ScAT and RpAT of AD and UN offspring as indicated by increased glycerol release (Figure 5A and B). An insulin-dependent decrease in lipolytic responses was observed in ScAT in both AD and UN offspring, whilst RpAT lipolytic responses were not influenced by insulin. The pattern of lipolytic response in either depot was not influenced by prenatal undernutrition.

## Discussion

The prevalence of obesity in Western and developing countries has increased to epidemic proportions. Obesity is a major risk factor for type II diabetes and cardiovascular disease, and leads to chronic illness causing reduced quality of life, increasing costs of medical care, and premature mortality. Adipose tissues have a strong influence on glucose and lipid metabolism, and it has been proposed that metabolic dysregulation in obese subjects may be related to changes in adipose tissue physiology [13]. Visceral adipose tissue has previously been identified as a major driver of metabolic disease processes [10, 14, 15]. Therefore, we investigated the role of specific adipose tissue depots in the pathogenesis of metabolic dysregulation in an animal model of metabolic programming. The present study showed that changes of glucose and lipid metabolism in adipose tissue may play a strong role in the perturbed metabolic regulation induced by maternal malnutrition during foetal development. Our data suggest that reduced insulin responses in abdominal adipose tissue depots, as shown here by significant changes in the structure and function of RpAT from offspring of rats undernourished during pregnancy, may directly contribute to changes in whole body energy metabolism.

### Adipose tissue responses to prenatal undernutrition

White adipose tissue compartments in the rodent develop during the later stages of pregnancy and visceral and subcutaneous tissue depots have recently been shown to have distinct developmental origins [8]. It has previously been shown that foetal adipogenesis and development of adipose tissue depots is influenced by maternal malnutrition. Therefore, we investigated whether nutritional variations during foetal development may lead to long-term changes in adipocyte physiology, potentially resulting in pathophysiological consequences such as insulin resistance and inflammation in later life [13, 16–18]. It has been argued that prenatal undernutrition during pregnancy may set in train a range of physiological adaptive processes which prepare offspring for a food-deprived environment during later life. The metabolic phenotype that develops is commonly described as a “thrifty” phenotype. It is hypothesised that offspring are physiologically adapted to store energy more readily in times where food is plentiful and are able to release this stored energy more readily when food is scarce [19]. This energy storage phenotype is clearly evident when UN offspring are fed a high fat hypercaloric diet during postnatal life, which then leads to overt obesity [6].

Our previous research has described that there are key differences in energy regulating pathways in the muscle and liver of UN offspring with different metabolic consequences [5–7]. Although there is growing evidence of major ontogenetic differences between visceral and subcutaneous adipose tissue [8], little is known about the influence of prenatal nutritional perturbations on these important contributors to homeostatic regulation of energy metabolism. We therefore investigated key physiological pathways of two distinct white adipose tissue depots in offspring of rats undernourished during pregnancy. Accumulation of visceral adipose tissue has previously been identified as a major driver of metabolic disease processes including insulin resistance and chronic inflammation [10, 14, 15]. The retroperitoneal adipose tissue (RpAT) examined in the present study belongs to the intra-abdominal, visceral fat depots, which are known to be enlarged by both diet- and prenatally-induced obesity [6, 20]. In contrast, subcutaneous adipose tissue represents fat stored directly under the skin. The subcutaneous adipose tissue (ScAT) depot is generally considered to play a lesser role in metabolic disease processes compared with the retroperitoneal tissue, and in some studies has been identified to support improved glucose tolerance [21].

In the present study, elevated plasma triglyceride concentrations and a larger adipocyte size observed in UN offspring suggest the presence of significant long-term metabolic programming of adipose tissue physiology through prenatal undernutrition during pregnancy. Both, the retroperitoneal and the subcutaneous adipose tissue depots of UN offspring contained larger adipocytes, even at a lower body weight in comparison with control rats. Increasing adipocyte size through enhanced triglyceride storage (hypertrophy) is a key feature in the growth of adipose tissue during obesity development [22]. This observation is supported by our previous studies where DEXA scanning results identified that UN offspring had a higher percentage of total body fat compared to AD controls [7]. Interestingly, the histological identification of larger adipocytes at a lower body weight in UN offspring suggests a constitutively higher fat accretion per cell and altered energy storage even in the absence of overt obesity. To further explore the biological nature of this metabolic setting, the present study investigated the responsiveness of different adipose tissue depots to endocrine stimulation by insulin and a beta_3_-adrenergic agonist in offspring of dams undernourished throughout pregnancy.

### Insulin responsiveness of adipose tissues

In the *ex vivo* studies, the basal glucose uptake in RpAT was similar in both AD and UN offspring. However, the glucose uptake stimulated by insulin was significantly lower in both adipose tissues depots of UN offspring compared with AD adipose tissues. The RpAT from UN offspring showed the smallest change in glucose uptake in response to insulin. This reduction in insulin sensitivity in RpAT was reflected by lower levels of the expression of the proteins of the insulin signalling pathway; InsRβ, PI3K, PKCζ and mTOR proteins were all present at significantly lower levels in RpAT of UN offspring. In support of this observation, a previous clinical study identified a decrease in key insulin signalling molecules in adipose tissue of young men who were of low-birth-weight [23]. The insulin signalling molecules InsRβ, PI3K, PKCζ and mTOR are involved in the regulation of adipogenesis and glucose uptake in mammalian cells [24, 25]. Our study therefore suggests that the reduced expression of these key components of the insulin signalling cascade in RpAT of UN offspring may be a plausible mechanism that could explain the reduced insulin response observed in this tissue.

In the ScAT explants, basal glucose uptake and expression of markers of the insulin signalling cascade were similar in both AD and UN offspring. However, the stimulation of glucose uptake by insulin was less pronounced in ScAT from UN offspring. The mechanism of this reduction in insulin responsiveness in this tissue depot remains to be elucidated [26]. However, whilst our present data cannot explain the adipocyte hypertrophy in ScAT from UN offspring, it is tempting to speculate that alterations in insulin signalling may play a key role in this process because hypertrophic adipocytes in humans have previously been linked with changes in gene expression related to insulin resistance [27].

### Adipocyte hypertrophy and metabolism

Adipocyte hypertrophy is the result of increasing storage of lipids in the singular fat vacuole of white adipocytes. Stored lipids within the vacuole stem from both *de novo* fatty acid synthesis from glucose and plasma fatty acids, which are taken up by the adipocyte. The extent of fat storage is counterbalanced by lipolysis. Lipolysis results in fatty acid release from the cell and consequently, a decrease of adipocyte size. Our results identified that basal and beta_3_-stimulated glycerol release, reflecting the rate of lipolysis in adipose tissues, was not influenced by prenatal nutrition; but the basal lipolysis rate was generally higher in RpAT than ScAT. As anticipated, insulin inhibited beta_3_-adrenergic receptor-induced lipolysis in ScAT, while in RpAT insulin could not diminish glycerol release stimulated by BRL reflecting the reduced insulin sensitivity observed in this adipose tissue depot discussed above. The observation that insulin’s inhibitory effect on lipolysis varies between tissue depots has previously been observed in chow fed mice and supports our research methodology [28]. We hypothesise, from these observations that reduced fatty acid release from adipocytes was not responsible for elevated adipocyte fat storage in these rodents because lipolysis in *ex vivo* tissue samples was not influenced by prenatal undernutrition. Furthermore, reduced insulin sensitivity in adipocytes of UN offspring, as observed in the present study, would decrease the rate of *de novo* fatty acid synthesis in these adipocyte cells due to a lack of ability to take up glucose as substrate. Therefore, we propose that the adipocyte hypertrophy might be based on a higher transfer of fatty acids to the adipose tissue from other tissues. The most likely physiological origin of fatty acids is the liver, which can synthesize fatty acids from excess circulating glucose [29]. This notion is in agreement with our previous report that UN offspring may have a higher hepatic capacity for *de novo* fatty acid synthesis as indicated by a significantly increased expression of hepatic fatty acid synthase mRNA levels [6]. This observation could also offer an explanation for the elevated plasma triglyceride concentrations in UN offspring observed in the present study. In addition, triglyceride storage capability of adipocytes may be a limiting factor in this setting.

Despite a reduced capacity to take up glucose directly in adipose tissues, there was no increase in plasma glucose concentrations in UN offspring. This could be explained by, and is in agreement with our previous studies, which showed that offspring of undernourished mothers have an increased capacity to store glucose as glycogen in liver and muscle [5–7, 12]. This observation contrasts the known changes in metabolic regulation observed during starvation and re-feeding. In adult rodents, re-feeding following a period of starvation results in a redistribution of insulin-stimulated glucose uptake away from muscle towards subcutaneous and retroperitoneal adipose tissues and consequently results in increased fat accretion [30]. It is possible that, in prenatally undernourished rats, an adipose tissue depot-specific reduction in insulin sensitivity may shift glucose to liver and muscle. In this setting, any excess glucose would be utilised for increased hepatic *de novo* synthesis of fatty acids. Importantly, the establishment of insulin resistance in RpAT might constitute a key pathway in the pathogenesis of metabolic dysregulation commonly observed during prenatally-induced obesity development.

In summary, maternal undernutrition during pregnancy caused clear adipose tissue depot-specific changes in insulin sensitivity and reduced glucose uptake in RpAT in offspring. The reduced insulin response in RpAT may reflect a change in energy storage and glucose utilization in adipose tissue [5], which might initiate a shift in glucose utilization from adipose tissue to liver and especially muscle [7]. Furthermore, these findings emphasize the important role of changes in endocrine responsiveness of visceral adipose tissue as one of the key steps in the development of metabolic dysregulation. The role of different adipose tissue depots in the pathogenesis of metabolic disorders requires further investigation in human clinical studies, especially in light of the recently described paradox of a metabolically healthy form of obesity and the long-term risk of cardiovascular disease [31].

## Authors’ information

Dr Nichola Thompson and Prof Korinna Huber share first authorship.

## Abbreviations

ScAT: Subcutaneous adipose tissue
RpAT: Retroperitoneal adipose tissue
UN: Prenatally undernourished
AD: Prenatally adequately nourished
BRL: Beta_3_-adrenergic receptor ligand.
